# Multiplex Lateral Flow Immunoassay: An Overview of Strategies towards High-throughput Point-of-Need Testing

**DOI:** 10.3390/bios9010002

**Published:** 2018-12-26

**Authors:** Laura Anfossi, Fabio Di Nardo, Simone Cavalera, Cristina Giovannoli, Claudio Baggiani

**Affiliations:** Department of Chemistry, University of Turin, Via Giuria, 5, I-10125 Turin, Italy; fabio.dinardo@unito.it (F.D.N.); simone.cavalera@unito.it (S.C.); cristina.giovannoli@unito.it (C.G.); claudio.baggiani@unito.it (C.B.)

**Keywords:** immunoassay, rapid test, point-of-care testing, immunochromatographic test

## Abstract

Simultaneous measurement of different substances from a single sample is an emerging issue for achieving efficient and high-throughput detection in several fields of application. Although immunoanalytical techniques have well-established and prevailing advantages over alternative screening analytical platforms, one of the incoming challenges for immunoassay is exact multiplexing. Lateral flow immunoassay (LFIA) is a leading immunoanalytical technique for onsite analysis, thanks to its simplicity, rapidity, and cost-effectiveness. Moreover, LFIA architecture is adaptable to multiplexing, and is therefore a possible answer to the pressing demand of multiplexing point-of-need analysis. This review presents an overview of diverse approaches for multiplex LFIA, with a special focus on strategies based on new types of magnetic, fluorescent, and colored labels.

## 1. Introduction

A lateral flow device (LFD) is a particular type of biosensor, in which the recognition layer is fabricated onto the surface of a porous membrane. The membrane creates and sustains the flow of the sample and reagents by capillarity, and holds specific recognition elements that are confined in spatially defined zones of the membrane itself, which are identified as reactive zones or detection sites. Usually, but not necessarily, the recognition elements are specific antibodies, and the biosensor relies on immunoassay principles, giving rise to the so-called ‘lateral flow immunoassay’ (LFIA), also called the immunochromatographic (strip) test. The signal generates from reactions that involve the sample, the antibodies, and a suitable probe, and takes place in the correspondence of the reactive zones of the membrane. To summarize, the membrane, the probe, and the antibodies used are equally important in determining the functionality of the biosensor.

**Figure 1:**
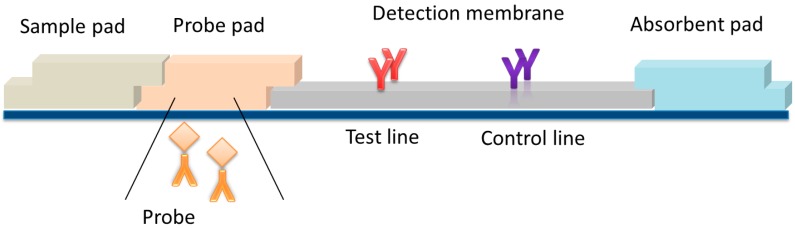
A typical lateral flow immunoassay (LFIA) strip: the strip architecture includes the detection membrane, where selective antibodies are aligned to form the test and control lines, additional membranes (i.e., the sample probe and absorbent pads), a rigid structure and the probe.

The architecture of a typical LFD includes: the active porous membrane, additional membranes (i.e., the sample, probe, and absorbent pads), and a structure that guarantees steadiness and consistency (Figure 1). As regards the probe, suitable bioselective reagents are linked to micro- or nano-materials that provide the signal and enable the detection of the reaction occurring on the membrane. Most LFDs rely on a visual readout. Therefore, conventional labels are colored micro- or nanoparticles.

The first lateral flow immunoassay (LFIA) was described and patented in the late 1970. A few years later, the LFD for ascertaining pregnancy (by measuring levels of the human hormone chorionic gonadotropin in urine) broke into the market, giving rise to a paradigmatic change of the concept of health care that led to the modern ideas of ‘point-of-care testing’ and, ultimately, of ‘personalized medicine’ [1,2]. Since then, LFIA has spread widely in clinical diagnostics, both as a low-tech robust assay for applications in remote and/or resource poor environments, and also for practitioners in day-to-day clinical work [3]. Meanwhile, the concept of ‘point-of-care’ testing has crossed the boundaries of medicine, so the general term ‘point-of-need testing’ is used to refer to most portable analytical systems that can furnish the response where the analytical demand is posed. Accordingly, LFIA technology has also found applications in diverse fields, where its inherent advantages of simplicity, rapidity, cost-effectiveness, and no requirement of equipment or technical expertise are crucial. Examples of the employment of LFIAs are found in veterinary, forensic, food safety, and environmental analysis. 

LFDs are usually developed for the detection of a single compound per assay. However, simultaneous on-site measurement of different substances from a single sample is becoming more and more important [4]. The capability of multiplexing has several benefits, including improving the efficiency of testing and reducing costs, and is strongly requested for those applications in which advanced decision-making is requested or availability of samples is limited. 

In order to improve the detection efficiency and achieve high-throughput detection, lots of efforts have been made on immunoassay, and in particular on LFIA. Multiplexing LFIA (xLFIA) has been realized through several approaches, which can be divided in two main groups: multiplexing strategies involving a modification of the architecture of the device and multiplexing strategies based on the use of suitable probes.

**Figure 2:**
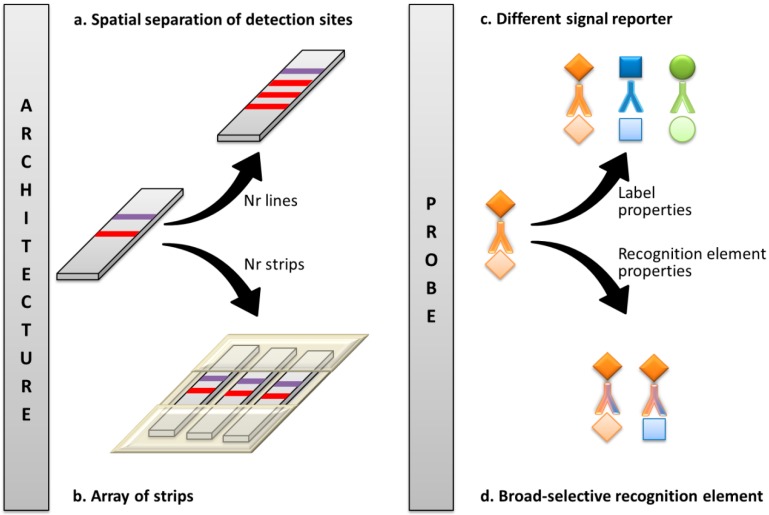
Strategies for multiplexing LFIA by: (**a**) spatially separate detection sites in a single strip; (**b**) aligning several strips in an array format; (**c**) exploiting signal reporters that provide different signals; and (**d**) using broad-selective antibodies able to bind to several compounds in a class.

Compared to other immunoanalytical techniques, such as those carried out in microplate format (i.e., ELISA, Enzyme Linked ImmunoSorbent assay), LFIA is particularly suited for enabling multiplex analysis. Indeed, the architecture of an LFD includes the possibility of aligning more than one detection site in a single analytical device (Figure 1). As a rule, a LFD includes at least two reaction sites, one that responds to the compound to be detected (called the test line), and a second reaction site that is used as an internal control to ensure correct operation of the device (control line) [5]. Binding events at the two reaction sites usually happen independently from each other, although involving one sample and one probe. Therefore, multiplexing can be conceived simply as appending further reaction sites along the length of the strip (provided that no reciprocal interference between the different reactions exists). The spatial separation of multiple detection sites requires the minimum manipulation of the assay architecture (Figure 2a) and is the far most popular way to achieve multiplexing in LFIA [6,7,8,9,10,11,12,13,14,15,16,17,18,19,20]. However, interpreting the qualitative results of a multi-zone lateral flow assay is not as simple as that of a conventional single-parameter assay. An increase in the number of analytes of interest interferes with the identification of the closely spaced test zones. Attempting to mitigate this issue by increasing the distance between these zones obviously leads to increased consumption of sample and membrane materials, as well as increased assay times [21,22].

Along with the spatial separation of detection sites in one strip, the separation of reaction sites using individual strips arranged in an ‘array-like’ format (Figure 2b) has also been widely exploited [22,23,24,25,26,27,28,29]. This second approach again impacts the architecture of the assay, with even lower constraints than the first. In particular, several assays are realized in parallel onto different strips that share only the sample. As a consequence, no risk of reciprocal interference exists between assays. Nevertheless, the costs of fabrication and the volume of the sample needed increase proportionally with the number of analytes being detected.

Strategies for multiplexing that have been developed primarily for other immuno-analytical techniques can be transferred to setting xLFIA. For instance, the development of antibodies, or other recognition elements that are group-specific to contaminants, can be used to develop detection methods for a class of structurally related compounds (Figure 2c) [30,31,32]. Moreover, labels that provide distinguishable signals can be exploited to individually detect different compounds in a single reaction site (Figure 2d) [33,34,35,36]. This last approach requires different labels that can be detected by one detection system, such as, for example, fluorescent labels that are excited by a single source while showing different emissions, or multi-chromatic labels. Several nanomaterials with suitable features (namely, quantum dots, QD, colored latex beads, lyposome-encapsulated dyes, etc.) have been used to design this kind of xLFIA. Nevertheless, they have been mostly employed to facilitate the identification of spatially separated detection sites, rather than for enabling multiple detections in a single reaction site.

**Figure 3:**
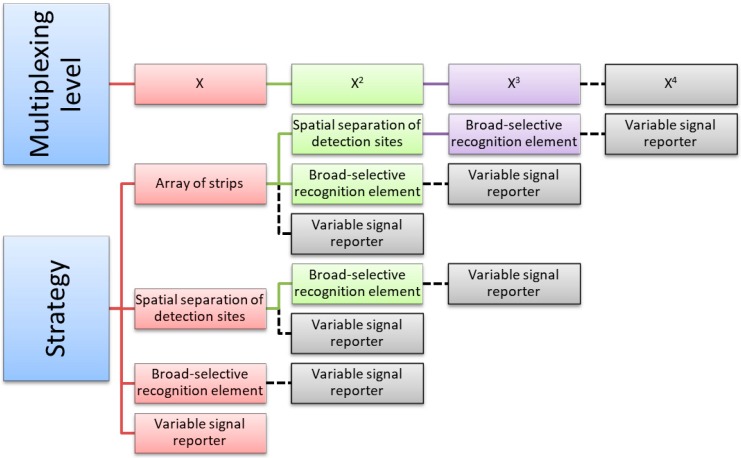
Approaches to multiplexing LFIA that can be exploited separately (single ‘x’ multiplexing level) or combining two (x^2^), three (x^3^) or even four (x^4^) of them. Combinations highlighted in colors have been reported in literature, while multiplexing strategies theoretically realizable but not yet reported are shown in grey.

All above described approaches are reciprocally independent and can be integrated in order to significantly enlarge the number of compounds that can be analyzed simultaneously by a single LFIA device (Figure 3). Conceptually, rolling two, three, and even all four multiplexing approaches in one device is a feasible way towards high-throughput analytical systems. However, this comes at the expense of simplicity and operability by untrained personnel, which is a distinct trait of the LFIA technique. The design of high-throughput multiplexing strategies should be balanced with guarantee of retaining the primary benefits of the technique, such as its rapidness, on-field applicability, simplicity of execution and detection, and cost-effectiveness. This review will discuss critically the approaches for multiplexing LFIA based on engineering (i) the architecture of the device, and (ii) the probe design. It will focus on perspectives in the use of new nanomaterials as efficient versatile labels for enabling xLFIA in a single strip.

Although LFA mostly uses antibodies as the specific recognition elements, the platform allows for the rapid and equipment-free detection of other bio-affinity interactions. The NALFIA (nucleic acid lateral flow immunoassay) technique employs the architecture of LFA devices and similar signal reporters, while the recognition element is represented by a DNA or RNA probe. A hybrid approach uses aptamers as the specific recognition element, instead of antibodies. Aptamer-based lateral flow assays are now emerging, due to the potential advantages of aptamers as compared to their antibody counterparts. Aptamers are produced in vitro, thus avoiding the use and sacrifice of animal hosts, lowering batch to batch variations, and allowing for the selection of aptamers in non-physiological conditions (that simulate conditions in which the aptamer will be applied). Immunogenicity is not required and the toxicity of the target compound does not hamper the obtainment of aptamers [37,38]. 

The development of synthetic recognition elements to replace antibodies where these are difficult to be obtained represents a viable way towards expanding LFA applicability. The multiplexing strategies that apply for LFIA can be transferred to other LFA techniques employing nucleic acids or synthetic recognition elements. Therefore, this review will focus on strategies for multiplexing lateral flow immunoassay.

Alternative approaches for multiple analysis exploiting paper-based assays have been described. Indeed, paper-based biosensors show multiplexing capabilities to various degrees, as recently reviewed by Li and Mcdonald [21]. However, among others paper-based devices for designing multiplexing point-of-care devices, the LFA platform shows higher versatility and readiness. Moreover, the more pressing demand for multiplexing arises from applications other than clinical diagnosis (such as, for example, for forensic and food safety controls, where the number of target compounds that should be detected in a single sample is quite large), which are prompting and directing the technological advances in the field.

## 2. Strategies for Multiplexing LFIA

### 2.1. One-Strip xLFIA: Spatial Separation of Detection Sites

The by far most popular strategy for multiplexing LFIAs is the design of several test lines or dots on an immunochromatographic strip using gold nanoparticles (GNP), quantum dots, or colored/fluorescence microspheres as labels. A shortcoming of fluorescence nanoparticles is that they require using an external device for the excitation, so colored nanoparticles that can be visualized by the naked eye have been investigated more extensively. Therefore, the approach based on drawing two or more lines in a single strip and using GNP as the label has been more largely reported [6,7,8,9,10,11,12,13,14,15,16,17]. In particular, this approach has been applied for the simultaneous detection of food contaminants, such as mycotoxins [6,13,16,17], antibiotics [8,9,12], and pesticides [10,14,15]. The number of lines that can be arranged in a single strip is limited, if not resorting to strip elongation. And strip elongation requires increasing the amount of sample to be employed and the time for completing the assay. Furthermore, reproducibility of the properties of the nitrocellulose membrane and, as a consequence of the flow, becomes triggering while increasing the strip length. The maximum number of lines that have been included in a single strip is six (comprising the control line), which means that up to five different compounds were detected simultaneously [8,14,15]. Most frequently, two-three analytes are detected by one-strip xLFIAs [6,9,10,11,12,13,16,17].

**Figure 4:**
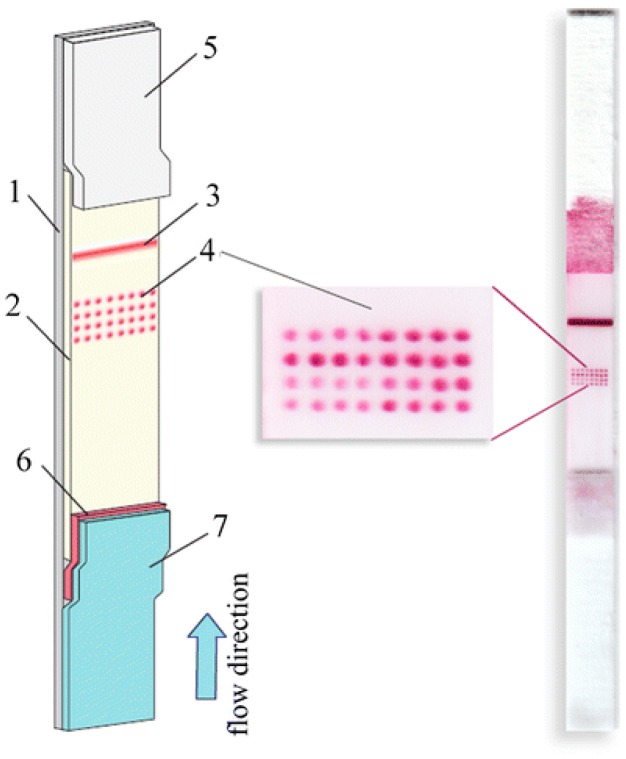
Schematic representation and image of the lateral flow microarray test strip. Each of the 32 dots represents a different capturing agent; the validity of the test is assured by the control line as usual [17].

A feasible way to vastly increase the number of spatially separated detection sites is the conversion of the detection site itself from line-shaped to dot-shaped. Indeed, the number of individual dots that can be arranged in a strip is far greater than the number of lines. As an example, the work of Taranova et al. reported the fabrication of a LFD in which the test zone of the nitrocellulose support comprised a microarray spotted with up to 32 capturing antigens (Figure 4). Correspondently, thirty-two different drugs of abuse were simultaneously detected in urine by means of the xLFIA [18]. The approach greatly improved productivity of the assay. However, this was at the expenses of the robustness of the assay result, because the smaller the detection site, the higher is the impact of flow inhomogeneity on the generation of the signal.

The arrangement of several dots in a single strip can also be exploited to design protein microarrays in a LFA platform. This approach has been designated ‘lateral flow microarray’ and was first reported by Gantelius et al. for the diagnosis of bovine pleuropneumonia in serum. In this work, the microarray comprised a panel of recombinant protein antigens arranged onto the membrane of a single LFA strip. The panel of antigens allowed for the diagnosing of the infection with higher sensitivity compared to diagnostics employing one or few antigens mixed in a conventional test line [19]. A similar approach was exploited by the same group for the diagnosis of allergies, through the creation of a protein microarray comprising 15 allergens [20]. Spots were obtained by dropping approximately 300 pL, resulting in a spot size of 120 mm. The array layout consisted of 15 rows of allergens with four downstream identical spots for each allergen. Each spot was separately quantified by means of a dedicated reader. Conveniently, the multiple spots enabled not only multiplexing, but also averaging data from identical spots (that compensated the larger variability of the deposition of very small volumes of reagents). However, the device required specialized equipment for data acquisition.

Microarray on LFA platforms have been reported more frequently for the detection of DNA and RNA sequences [39,40]. Although these applications cannot strictly pertain to the lateral flow immunoassay technique, nucleic acid LFA microarrays trace feasible routes for the development of LFIAs in the microarray format, too.

### 2.2. Array of Strips

**Figure 5:**
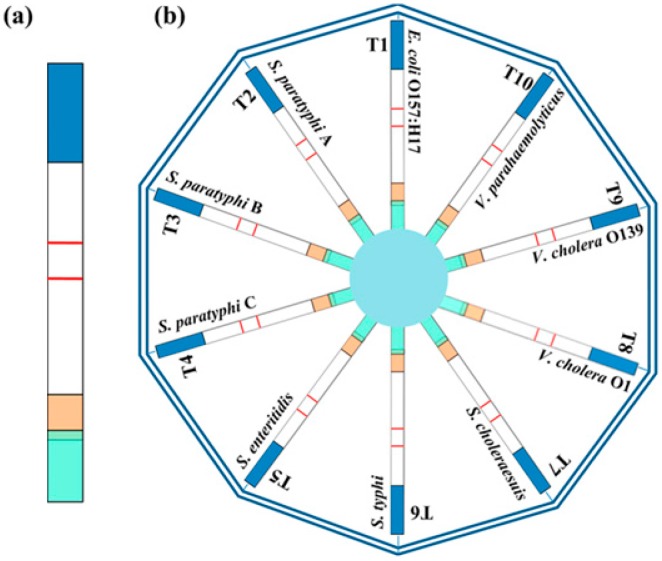
(**a**) Individual strip for the identification of a single bacterium by means of an antibody linked to the up-converting phosphorous probe. (**b**) The 10-channel up-converting phosphor technology-based lateral flow (TC-UPT-LF) disc holds 10 detection channels (T1 to T10), each comprising a single strip for the target bacteria [23].

Several individual strips can be arranged in a special holder, also designed as to collect one sample and distribute it between several strips. The sample flows in parallel on each strip, which in turn can hold one or more test lines. The advantages of this approach rely on the large multiplexing capability and on the absence of reciprocal interference between assays that happen independently on individual strips. Indeed, each assay can be developed singularly and then put together, without requiring any further optimization [22]. As an illustration of the multiplexing ability of the ‘array of strips’ approach, Hong et al. demonstrated the simultaneous detection of 10 different antibodies by using a 10-channel up-converting phosphor technology-based lateral flow (TC-UPT-LF). In that study, after optimization one by one, each strip was integrated into a TC-UPT-LF disc to profile antibodies against *Yersinia pestis*. Similarly, the 10-channel disc configuration was employed to simultaneous detect ten foodborne pathogens (Figure 5) [23]. The ‘array of strips’ approach is popular among manufacturers and is commonly found in drug of abuse testing devices [24,25,26,27].

LFDs implementing the ‘array of strips’ strategy are well suited for applications in which large volumes of samples are available. Usually, each strip requires 50–100 µL of liquid to complete the assay and the amount of sample required increases when the number of strips increases. In fact, these kinds of LFDs are used for the onsite rapid detection of urinary and salivary metabolites [24,25,26,27], thus applies to biological fluids that can be collected non-invasively. Similarly, their usage can be envisaged for high-throughput analysis in food safety assessment, where the amount of sample available is usually not a restraint. 

Besides requiring large volumes of the sample, the ‘array of strips’ approach is also costly compared to other multiplexing strategies employing the same bio-reagents and probes. Costs of LFD production include materials and chemicals used to prevent matrix interference and to guarantee long-term stability. Therefore, the higher the number of strips, the higher the cost of the device.

A tentative option to overcome limitations due to sample availability and cost of production has been proposed by Schenk et al., who described the first steps aimed at developing a ‘multi-channel strip’ for the simultaneous detection of the lipopolysaccharides of *Salmonella typhimurium* and *Salmonella enteritidis* [28]. In their work, the authors structured channels in the nitrocellulose membrane by means of laser ablation. In this way, four distinct tracks of porous material were formed in the width of a usual strip, and each of the channels was operated as a distinct strip. Preliminary, studies on the wicking properties of the laser ablated nitrocellulose showed that a minimum channel width and a minimum barrier thickness between channels are required for obtaining reproducible flowing [29]. Although these works represent an interesting attempt at expanding the multiplexing capability of the one-strip LFIA, the readability of the assay result should also be carefully considered when miniaturizing point-of-need testing systems that are meant to be used by untrained operators.

### 2.3. Multiplexing LFIA Based on the Probe

Here, the probe is a conjugate between a recognition element, which is a moiety able to bind to reagents forming test and control lines and to the analyte, and a label, that generates a detectable signal (also referred as the signal reporter). Adapting both the recognition element and the signal reporter allows multiplexing (Figure 3c,d).

In one sense, broad-specific recognition elements have been developed with the aim of detecting several compounds in a group or a class. As a matter of fact, class-selective antibodies have been prepared and used to measure several analytes (within a class of compounds) by means of LF devices: Zhang et al. developed a monoclonal antibody (mAb), which was able to recognize three major ochratoxins, and based on this mAb, they proposed a LFIA for the simultaneous detection of the three hazardous substances [30]. Similarly, Xie et al. generated a monocolonal antibody that equally recognizes avermectin and ivermectin and employed it for setting up a dual LFIA [31]. Notably, Wang R. et al. reported a xLFIA for measuring up to 7 β-agonists in a single run by using a monoclonal that recognized clenbuterol and its analogues [32]. 

However, this approach needs complicated processes for the production of the antibodies and is confined to applications in which the useful information is the presence of any of the compounds in the class (or their sum), rather than the identification of one specific compound, which significantly reduces their practical use.

The use of various labels (e.g., enzymes, fluorophores, and nanoparticles) can be regarded as a viable alternative for xLFIA multiplexing. Indeed, the exploitation of labels providing distinguishable signals allows differentiating between various complexes that are formed at the same site (i.e., at a single test line). This approach has seldom been used [33,34,35,36]. Wang W. et al. proposed a smart multiplexing strategy, based on the different kinetics of horseradish peroxidase and alkaline phosphatase to obtain a time-resolved chemiluminescence detection [33]. Accordingly, two antibodies (selective to ractopamine and clenbuterol, respectively) were mixed to form a single test line and the presence of any one of the two analytes was revealed by the same chemiluminescence signal, while the time of the signal generation allowed for distinguishing among the analytes. Similarly, Wang C. et al. exploited two QD emitting at different wavelengths as distinguishable labels and used them to tag specific antibodies directed towards two tumor markers in a the sandwich-type immunoassay. The captured antibodies were placed onto the nitrocellulose membrane to form a single test line and the two tumor markers were recognized by the color of the QD photoluminescence [34]. 

Multiplex detection in the ‘single test line’ format associated to colorimetric detection has also been described [35,36]. In the approach proposed by Yen et al., trichromatic silver nanoparticles were exploited for the simultaneous detection of different viruses by a sandwich xLFIA [35]. In particular, orange, red, and green silver nanoparticles were employed to set a xLFIA. However, the color of the mixed signal reporters made it almost impossible to evaluate the co-occurrence of analytes by the naked eye. Nevertheless, the recognition of viruses was made possible by digitally processing the images of the strips. 

**Figure 6:**
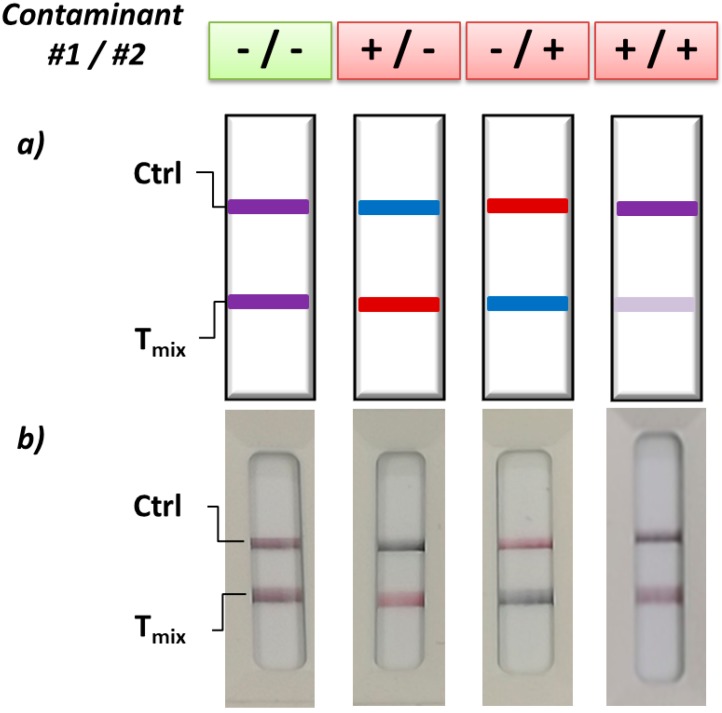
The xLFIA realized through the use of a single test line and multicolor gold nanoparticles (GNPs) as signal reporters. Red and blue GNPs were linked to antibodies directed towards two food contaminants and the test line was formed by the mixture of the two corresponding antigens. As a result of the immunoreactions occurring at the test zone, the test line assumed different colors accordingly to which contaminant was present in the sample. (**a**) Color code for contamination detection and contaminant identification. (**b**) Images of strips obtained by the color-encoded xLFIA [36].

Despite attracting attention, the integration of probes providing variable signals as a function of the analyte to be detected, has shown some limitations in the potential application for on-field analysis. In particular, the need for certain equipment to elaborate the assay result increases costs and limits viability. In an attempt to mitigating this weakness, our group developed a dichromatic xLFIA that used blue and red gold nanoparticles as the labels [36]. We prepared GNP that differed in shape (and therefore in the wavelength of the surface plasmon resonance peak) to tag two antibodies selective for two major mycotoxins (aflatoxin B1 and fumonisin B1), respectively, and developed an xLFIA in the competitive format to simultaneously detect the two toxic compounds. Antigens of each of the mycotoxins were mixed and dropped onto the nitrocellulose membrane to create a single test line. As a result of the simultaneous binding of the red- and blue-labelled antibodies to the mixed antigens, the test line appeared purple. Upon addition of one mycotoxin, the binding of the corresponding red-(blue) labelled antibody was inhibited at the test line, which thus appeared in the complementary color, namely blue (red). The simultaneous presence of both contaminants produced the complete disappearance of the test line, due to the contemporary inhibition of the binding of both red- and blue- labelled antibodies (Figure 6). Therefore, the contamination due to each mycotoxin was visually detected and discriminated based on a pre-defined color code. In this case, digital processing of images also enabled (semi-)quantitative analysis. A further benefit of using labels that show primary colors, besides facilitating the visual reading of results, can been seen in the simplicity of image processing through RGB data analysis.

In summary, the search for new labels that are able to provide variable signals represent a chance to expand the multiplexing capability of LFIA, especially if in combination with other multiplexing strategies. However, the realistic usage of new probes is strictly dependent on their easily integration into the LFIA systems (i.e., ability to freely flow through porous membranes, absence of non-specific interaction with typical LFD materials, etc.). Furthermore, new probes are required to guarantee simplicity and rapidity to the LFIA detection step.

### 2.4. x^n^LFIA: Integration of ‘Multiple’ Multiplexing Strategies in a Single LFIA Device

Ideally, each multiplexing approach described above can be combined with others to enhance further the productive potential of LFIA.

The integration of two (x^2^LFIA) and, sometimes, three different strategies (x^3^LFIA) was reported, enabling high-throughput analysis. For instance, dozens of compounds were detected within minutes, requiring only a few microliters of the sample and no equipment. 

In this sense, broad-selective antibodies have been used in combination with the manipulation of the device architecture for the detection of antibiotic families in milk [41,42]. Wang C et al. used three class-selective antibodies and latex beads and colloidal gold as colored reporters to detect twelve sulfonamide, eighteen quinolone, and six tetracycline residues by a single LFD [41]. In this work, multicolored labels were employed to simplify the identification of the detection sites and did not contribute to further increasing multiplexing. Han et al. also exploited group-selective antibodies for measuring three classes of antimicrobial agents combined with employing GNP as the label [42]. In these examples, the spatial separation of detection sites and the broad-specificity of antibodies were employed as orthogonal resources (x^2^LFIA) for expanding the multiplexing quality of the device. As a result, they achieved the ability of simultaneously detecting up to thirty-six compounds by a single device and using just few microliters of sample. 

Multiplex LFIA realized through the spatial separation of lines in one strip has also been combined with the exploitation of innovative labels, such as up-converting phosphor reporter particles [43,44], magnetic nanobeads [45,46], enzymes triggering chemiluminescence [47], and near-infrared fluorescence labels [48]. In the last paper, the detection of three classes of antimicrobial agents in a single run was achieved by combining the use of three broad-specific antibodies and the spatial separation of three lines in one strip. Therefore, a x^2^LFIA was realized, increasing the total number of target compounds that were detected by a single device. As a further advantage, the spatial separation of detection sites allowed for individually detecting the three classes of antibiotics, thus enabling their recognition. 

Most devices in the array format not only incorporate several strips, but each strip is designed for the detection of more than one compound. In particular, LFD for detecting drugs of abuse are designed in the ‘array of strips’ configuration, in which individual strips show several spatially separated detection sites and, in some cases, also use class-selective antibodies. The combination of the three strategies (x^3^LFIA) gave rise to LFDs that are able to simultaneously detect and identify up to 20 different compounds, with limited requirements for increasing the sample volume [25].

## 3. New Labels for xLFIA

Considering that LFDs are intended for operation by untrained personnel, often multicolored labels have been used to facilitate distinguishing the various detected analytes. In particular, spatially separated detection sites were identified, not only based on positioning of the line along the strip, but also on the specific color of the probe used [41,49,50,51,52,53,54,55,56].

The same intent of simplifying analyte identification by means of labels with different optical properties has been pursued in several works based on the use of quantum dots (QD) [20,52,53,54,55]. Reportedly, QD are ideal labels for xLFIA, as they emit light at different wavelengths with narrow and intense emission peaks, while the excitation band is wide. Therefore, differently-colored emissions are obtained upon excitation by a single light source. Despite this beneficial characteristic, QD suffer from several drawbacks that have limited their wide employment as labels for xLFIA, such as: low hydrophilicity, lack of functional groups for conjugation to biomolecules (and especially to recognition elements), poor long-term stability, and strong non-specific interactions with materials typically use in LFDs [56]. Attempts have been made to overcome some of these limitations, including coating QD with amphiphilic polymers or encapsulating them in silica nanoparticles. These strategies permitted the introduction of chemical functionalities to QD and increase their stability and water compatibility. However, this comes at the expenses of their fluorescence quantum yield [57]. Furthermore, QD are made from toxic heavy metals, so they are being progressively replaced by fluorescent carbon nanomaterials (namely, carbon quantum dots, CQD), which are organic nanoparticles with similar optical properties and less toxicity compared to inorganic QD. Additionally, CQD are hydrophilic and can be prepared as stable aqueous suspensions. They are synthesized from various carbonous precursors [58]. Therefore, CQD with intrinsic chemical functionalities suitable for their conjugation to biomolecules can be obtained from appropriate precursors [59,60]. The first xLFIA exploiting CQD as the label has been described by Fang et al. [61]. In their work, the authors developed a fluorescence LFD for detecting three tumor markers in a single analysis, in which three lines were drawn in one strip and multicolor CQD were used as labels for recognizing the different biomarkers. Again, the multi-chromaticity served for identification purposes rather than for enlarging the multiplexing quality of the device. Nevertheless, this new class of fluorescent nanomaterials is very promising as labels for future xLFIA development.

Magnetic nanolabels have permitted the development of multiplex methods that cannot be realized with colored or florescent labels. The potential of magnetic nanoparticles (MNP) as labels derives from the fact that they, unlike optical labels, can be easily detected inside 3D opaque porous structures, such as within the thickness of the LFIA membrane. The opportunity for measuring not only the portion of nanolabels that is located on the surface, but the whole amount of labels distributed in the width of the membrane, brings a significant increase in the assay sensitivity. Based on this principle, Lu et al. prepared immuno-magnetic probes by covalently linking MNP to specific antibodies directed towards two tumor markers. The immuno-magnetic probes were included in a dual LFD also comprising a single strip with two spatially separated test lines. The LFD permitted the simultaneous detection of the two biomarkers with high diagnostic sensitivity and specificity [45]. An original quantification of MNP by non-linear magnetization proposed by Nikitin at al. allowed for further the increase of the sensitivity of detection of immuno-magnetic assays [46]. The prospect of this novel quantification method was illustrated by four different rapid bio-sensing systems, including an xLFIA for the simultaneous detection of three model compounds. Also there, three spatially separated test lines were created on a single strip and the MNP were used as the label for quantification. Benefits of MNP in terms of increased sensitivity are counterbalanced by the requirement of some external devices for detection and, therefore, should be seriously considered, especially for onsite and low-resource setting applications.

## 4. Conclusions

The rapid evolution of lab-based analytical techniques, in particular the great technical improvements in liquid chromatography coupled to tandem mass spectrometry systems, is posing a new challenge to analytical methods traditionally employed as first level screening methods, such as those using biosensors. Massive sample processing and intrinsic multiplexing capability are making lab-based techniques very attractive, also because accurate and precise results are obtained in a reasonable time and with acceptable costs. Therefore, the incoming progress of instrumental techniques represents a threat to the predominance of immuno-analytical techniques, especially when several data are required to reach meaningful information. Increasing multiplexing ability is then a need, rather than a possibility, for the future of immunoassay.

Attempts aimed at multianalyte detection in microplate-based immunoassay have been described, which exploit all approaches above discussed. However due to the requirement for a specific instrumental set-up for quantifying the output of the assays, their application remains unpractical. Conversely, the LFIA platform is particularly adaptable for multiplexing purposes, hence the research is tremendously active in this field and several solutions enabling the simultaneous detection of few to dozens of compounds have been proposed so far. Strategies described are diverse and range from simply juxtaposing several strips in a multichannel holder to more sophisticated approaches based on new signal reporters. However, considering the demand for increasing productivity of biosensors in general and, of LFIA in particular, the number of applications is destined to grow further in the near future.

The exploitation of innovative signal reporters combined with technological advances that make ‘instrumental detection’ feasible through generic and widely available devices (such as smartphones) opens new perspectives to the field. Contemporaneous efforts should be devoted to harmonizing protocols and sample preparation for enabling the parallel execution of different assays and for minimizing production costs.

Finally, the scope of expanding multiplexing capability cannot be pursued without preserving those characteristics that make the LFDs best suited for fitting ASSURED (affordable, sensitive, specific, user-friendly, rapid and robust, equipment-free and deliverable to end-users) criteria [1] and that have designated as responsible for their current success and their expanding growth.
